# Chian Turpentine in Cancer

**Published:** 1880-08

**Authors:** 


CHIAN TURPENTINE IN CANCER.—Anything that has been proven by experience to have even materially ameliorated or lessened, the suffering of a cancerous patient, should claim the attention of the medical journalist, and such statements of facts as may have come under his observation, be laid before his readers. So many cancer cures, however, have strutted their brief hour upon the stage of public faith and confidence, to be relegated soon to the obscurity whence they came, that we feel chary in mentioning a new candidate for public favor, unless by the sanction or endorsement of a prominent name. But Professor John Clay, Obstetrical Surgeon to the Queen's Hospital, Birmingham, England, gives to the "Lancet" *his* experience with the article we have named, and from his reports of its splendid results in several cases of cancer of the uterus, there seems to remain no doubt of its great value and efficiency in carsinomitous affections of the female generative organs. He says: judging by my experience, it is no figurative expression to say that it acts as a direct poison upon the growth, probably causing its ultimate death." He uses the "solution of chian turpentine," which is prepared as follows, viz: "an ounce of chian turpentine is dissolved in two ounces of pure sulphuric ether; the solution is readily produced. To half an ounce of this solution is added, four ounces of solution of tragacanth, an ounce of simple syrup, forty grains of flowers of sulphur and water to sixteen ounces, to form an emulsion; dose, one ounce, three times daily. "The maximum dose of the chian turpentine which can be safely and continuously given, is twenty-five grains daily."

As the efficacy of the chian turpentine is probably in no wise dependent upon the articles associated with it in the mixture, it would, doubtless, be as well taken in a more simple and convenient shape, and conformably to the taste of the patient, as it requires to be used for weeks, and even months, to obtain its salutary effects.

